# Assessing key clinical parameters before and after intraventricular hemorrhage in very preterm infants

**DOI:** 10.1007/s00431-020-03585-9

**Published:** 2020-01-28

**Authors:** Renée Lampe, Esther Rieger-Fackeldey, Irina Sidorenko, Varvara Turova, Nikolai Botkin, Laura Eckardt, Ana Alves-Pinto, Andrey Kovtanyuk, Michael Schündeln, Ursula Felderhoff-Müser

**Affiliations:** 1 School of Medicine, Klinikum rechts der Isar, Orthopedic Department, Research Unit for Pediatric Neuroorthopedics and Cerebral Palsy of the Buhl-Strohmaier Foundation, Technical University of Munich, Ismaningerstr 22, 81675 Munich, Germany; 2 School of Medicine, Klinikum rechts der Isar, Department of Pediatrics, Technical University of Munich, Ismaningerstr 22, 81675 Munich, Germany; 3 Mathematical Faculty, Chair of Mathematical Modelling, Technical University of Munich, Boltzmannstr. 3, 85748 Garching, Germany; 4 University Hospital Essen, Department of Pediatrics I, Neonatology, Pediatric Intensive Care, Pediatric Neurology, Department of Pediatrics III, Pediatric Oncology, University Duisburg-Essen, Hufelandstraße 55, 45147 Essen, Germany

**Keywords:** Intraventricular cerebral hemorrhage, Preterm infants, Immature brain, Mean arterial pressure, Arterial carbon dioxide pressure, Cerebral blood flow

## Abstract

Intraventricular cerebral hemorrhage (IVH) is one of the most severe complications of premature birth, potentially leading to lifelong disability. The purpose of this paper is the assessment of the evolution of three of the most relevant parameters, before and after IVH: mean arterial pressure (MAP), arterial carbon dioxide pressure (pCO_2_), and cerebral blood flow (CBF). Clinical records of 254 preterm infants with a gestational age of 23–30 weeks, with and without a diagnosis of IVH, were reviewed for MAP and arterial pCO_2_ in the period up to 7 days before and 3 days after IVH or during the first 10 days of life in cases without IVH.

*Conclusion*: A statistically significant increase in pCO_2_ and decrease in MAP in patients with IVH compared with those without were detected. Both the mean values and the mean absolute deviations of CBF were computed in this study, and the latter was significantly higher than in control group. High deviations of CBF, as well as hypercapnia and hypotension, are likely to contribute to the rupture of cerebral blood vessels in preterm infants, and consequently, to the development of IVH.

**Table:** 

**What is Known:** • *The origin of IVH is multifactorial, but mean arterial pressure, carbon dioxide partial pressure, and cerebral blood flow are recognized as the most important parameters.* *• In premature infants, the autoregulation mechanisms are still underdeveloped and cannot compensate for cerebral blood flow fluctuations.*	
**What is New:** • *The numerical simulation of CBF is shown to be a promising approach that may be useful in the care of preterm infants.* *• The mean values of CBF before and after IVH in the affected group were similar to those in the control group, but the mean absolute deviations of CBF in the affected group before and after IVH were significantly higher than that in the control group.*

**What is Known:**

• *The origin of IVH is multifactorial, but mean arterial pressure, carbon dioxide partial pressure, and cerebral blood flow are recognized as the most important parameters.*

*• In premature infants, the autoregulation mechanisms are still underdeveloped and cannot compensate for cerebral blood flow fluctuations.*

**What is New:**

• *The numerical simulation of CBF is shown to be a promising approach that may be useful in the care of preterm infants.*

*• The mean values of CBF before and after IVH in the affected group were similar to those in the control group, but the mean absolute deviations of CBF in the affected group before and after IVH were significantly higher than that in the control group.*

## Introduction

Intraventricular cerebral hemorrhage (IVH) is the most frequent cause of brain damage in preterm infants [1, 2], leading frequently to neurodevelopmental disorders. In particular, preterm cerebral hemorrhage is regarded as one underlying cause of cerebral palsy [3–5]. IVH occurs with a frequency of 20–25% [1, 2] in preterm born infants with a gestational age (GA) < 32 weeks and/or a birth weight (BW) < 1500 g. It occurs most often in the subependymal germinal matrix [6, 7], a highly vascularized neuroepithelial structure with fragile vessels, which remains a part of the developing brain until the 30th week of gestation (WG). The germinal matrix vessels have a larger diameter than in the cortex, and therefore, a larger wall tension, and consequently, are likely to rupture leading to cerebral hemorrhage.

The origin of IVH is multifactorial [3], but arterial blood pressure, arterial carbon dioxide partial pressure, and cerebral blood flow are recognized [8–10] as the most important parameters. In the mature brain, intact cerebrovascular autoregulation ensures stable cerebral blood flow (CBF), even upon fluctuations in blood pressure [11]. In premature infants, however, autoregulation mechanisms are still underdeveloped and cannot compensate for these fluctuations [10, 12]; therefore, the rupture of fragile germinal matrix vessels can be triggered by ischemia or hyperperfusion [8, 9].

Regular monitoring of CBF level as well as observation of CBF fluctuations caused by pressure-passive cerebral circulation is crucial in preventing the occurrence of complications in preterm infants. Although several non-invasive techniques for measuring CBF, like transcranial Doppler ultrasound [13, 14] and near-infrared spectroscopy [15], exist, neither of these techniques is used as routine clinical procedures. Numerical assessment of CBF seems to be a promising approach in routine care of preterm infants. When relevant clinical parameter data, e.g., gestational age, birth weight, carbon dioxide partial pressure (pCO_2_), and mean arterial pressure (MAP), are fed into the model, the CBF can be immediately calculated and analyzed.

Despite numerous studies on the development of IVH, the focus of research has not been the evolution of relevant parameters such as pCO_2_, MAP, and CBF, before and after IVH. Such an analysis may, however, reveal to which extent some parameters can indicate a developing hemorrhage. In the current work, pCO_2_ and MAP were retrospectively obtained from standard clinical records of 254 very preterm infants. Values of CBF were estimated via a mathematical model recently developed by the authors for calculation of CBF [16–18]. Since initial conditions were similar, the evolution of parameters in the group of infants with a diagnosis of IVH was compared with equivalent values obtained from a group of preterms without a diagnosis of IVH.

## Materials and methods

The study was approved by the ethic committee of School of Medicine Klinikum rechts der Isar, Technical University of Munich (Ref. 364/15), and ethic committee of University Hospital Essen, University Duisburg-Essen (Ref. 16-7284-BO). No written patient consent was necessary for this retrospective study because of the following: (1) this report does not contain any personal information that could lead to the identification of the patient; (2) all data analyzed were collected as the part of routine diagnosis and treatment; (3) patient medical care was not set up for research purposes, but was the part of standard clinical procedure; (4) all patients were diagnosed and treated according to national guidelines and agreements.

Clinical data were collected from 254 preterm infants, which were born and treated postpartum at the neonatal intensive care units of two different university hospitals (Technical University Munich, School of Medicine, Klinikum rechts der Isar, and University Duisburg-Essen, University Hospital Essen). The data collected retrospectively covered a period of 11 years (2006–2016) in both hospitals. In both clinical centers, IVH was diagnosed by standard cranial ultrasound performed routinely on days 1, 3, 7, and 14 of life and more frequently (up to daily) in case of discrepancies or suspected hemorrhage. Examinations were carried out and classified by senior neonatologists. Higher IVH grades correspond to more severe hemorrhages according to Papile classification [19]. There were no exclusion criteria during data collection: all patients with IVH born in the time period from 2006 to 2016 were included. Depending on IVH diagnosis, infants were included either in the control group (no IVH) or affected group (with IVH).

MAP and pCO_2_ records were retrieved from routine clinical data collected during the first 10 days of life in the control group, and for up to 7 consecutive days before and 3 days after hemorrhage in the affected group. In order to increase the performance of statistical analysis of the study, both arterial and capillary blood gas values were considered. No distinction between two measuring methods was done because a strong correlation between arterial and capillary pCO_2_ (*r* = 0.96, *p* < 0.0001) was detected [20]. Although MAP and pCO_2_ were measured at different times and intervals, only coincident records of MAP and pCO_2_ were included in the analysis. The number of measurements per patient varied from 5 to 54, and the total number of coincident records of MAP and pCO_2_ was 3240.

For each clinical record of MAP and pCO_2_, the corresponding CBF value was calculated using a mathematical model adjusted for the immature brain of preterm infants [17, 18] from a model previously proposed for the adult brain [16]. The model takes into account anatomical and physiological parameters of cerebral circulation in the immature brain as realistically as possible. In the model, the vascular system is divided in 19 levels (9 arterial, 9 venous, and 1 capillary compartment) according to the morphological characteristics of the vessels. The presence of the germinal matrix is simulated according to GA by including an additional capillary network, parallel to the permanent network of vessels at the capillary level, and characterized by a realistic number and geometry of vessels. CBF is derived from the Kirchhoff’s law, in which the total resistance of the whole cerebral vessel network is computed as a sum of resistances of every compartment. The volume of blood flow depends on the perfusion pressure and vessel resistance, which in turn is influenced by the density and morphology of the vessel network and the rheological characteristics of blood. The dependence of vessels’ diameter on pCO_2_ is accounted for in the simulations by a reactivity mechanism which enables the extension or constriction of blood vessels depending on positive or negative deviation of pCO_2_ value from a nominal reference value. Simulation of the myogenic response to fluctuations in MAP is based on fittings to clinical data collected from preterm infants [18].

In addition, the mean absolute deviations of CBF (MAD_CBF_) were analyzed and compared. The MAD_CBF_ was calculated by taking the absolute value of the difference between the CBF value computed for each measurement and the mean of the control group for every gestational week.

Statistical analysis was performed by the comparison between the control and affected groups. Categorical characteristics were compared by their relative values in percent along with *p* value calculated by Fisher’s exact test. For continuous variables, mean values were compared using the two-sided Wilcoxon’s rank-sum test. A *p* value < 0.05 was considered to be statistically significant. Statistical analysis of the clinical records of MAP and pCO_2_ as well as mathematically calculated CBF was done for each gestational week and included comparisons between control and affected groups for time periods before and after IVH diagnosis. Children for which IVH was diagnosed on the first day of life were not considered in the analysis of parameters before IVH. In cases of IVH diagnosed on the second day of life, the period before IVH was limited to the first day of life. For infants with IVH diagnosis on the third day and onwards, the time period before IVH included the days preceding the day of the last routine ultrasound examination (e.g., only first day of life when IVH was diagnosed on the third day, and first 3 days of life when IVH was diagnosed on the fourth day). In cases when IVH was diagnosed after 10 days of life, only records collected during the first 10 days of life were taken into analysis. For the analysis of parameters following IVH, all medical characteristics were averaged over 3 days after the day of IVH diagnosis. In the control group, medical characteristics were averaged over 10 days. Presented below are the mean ± standard deviations of clinical parameters together with the *p* value associated to statistical tests. The analysis of the variance was made using box plots and the percentage of records located above the upper and below the lower threshold values. The latter values were evaluated from the group of infants without IVH as mean value ± standard deviation. All data were analyzed using the program MATLAB R2018b.

## Results

The cohort consisted of 254 very preterm infants with a GA between 23 and 30 weeks gestation (26.5 ± 2.1) and a BW between 335 and 1580 g (864.1 ± 279.1 g). A total of 136 patients were diagnosed with IVH grade I–IV (affected group), and 118 patients had no IVH (control group). The control group was matched with the affected group for GA and mean BW. Tables 1 and 2 contain information on the obstetric characteristics, the day of IVH diagnosis, and the grade of IVH.

**Table 1:** Obstetric characteristics of the cohort

Parameter		Control group 118 (100%)	Affected group 136 (100%)	*p* value
Gestational age		26.7 ± 2.2	26.3 ± 2.0	0.1
Birth weight		850.7 ± 252.8	875.7 ± 300.5	0.7
Male		48 (40.7%)	74 (54.4%)	0.03
Multiple birth		43 (36.4%)	52 (38.3%)	0.8
Parameters regarding pregnancy				
In vitro fertilization (IVF)		17 (14.4%)	15 (11.0%)	0.5
Chorioamnionitis/amniotic infection syndrome		50 (42.4%)	67 (49.3%)	0.3
Preterm premature rupture of membranes (PPROM)		39 (33.1%)	34 (25.0%)	0.2
EPH gestosis/preeclampsia		18 (15.3%)	7 (5.1%)	0.01
Parameters regarding the child				
Sepsis		50 (42.4%)	70 (51.5%)	0.2
Respiratory distress syndrome (RDS)		42 (35.6%)	42 (30.9%)	0.5
Pulmonary hemorrhage		5 (4.2%)	16 (11.8%)	0.04
Pulmonary stenosis		3 (2.5%)	1 (0.8%)	0.3
Acidosis	Respiratory	1 (0.9%)	0	0.5
	Metabolic	0	23 (16.9%)	< 0.01
	Respiratory + metabolic	5 (4.2%)	4 (2.9%)	0.7
Erythrocyte blood transfusion		71 (60.1%)	93 (68.4%)	0.2
Thrombocytopenia		8 (6.8%)	13 (9.6%)	0.5
Disseminated intravascular coagulation (DIC)		0	2 (1.5%)	0.5
Intrauterine growth retardation (IUGR)		8 (6.8%)	6 (4.4%)	0.4
Feto-fetal transfusion syndrome (FFTS)		4 (3.4%)	4 (2.9%)	1
Neonatal bowel perforation (spontaneous/focal intestinal perforation SIP/FIP)		3 (2.5%)	20 (14.7%)	<0.01
Necrotizing enterocolitis (NEC)		8 (6.8%)	12 (8.8%)	0.6
Cholestasis		1 (0.8%)	11 (8.1%)	0.01

**Table 2:** Number of premature infants for different weeks of gestation, grades of IVH, and the day of IVH diagnosis

WG	IVH		IVH Grade				Day of IVH diagnosis					
	No	With	I	II	III	IV	1st	2nd	3rd	4th	5th	> 5th
23	9	17	2	6	9	-	3	2	7	1	1	3
24	22	24	3	10	9	2	3	3	9	6	-	3
25	17	23	7	8	5	3	3	4	7	5	1	3
26	17	20	4	6	8	2	-	3	10	2	3	2
27	12	15	7	2	5	1	4	1	1	3	2	4
28	16	18	7	5	6	-	2	5	4	1	1	5
29	12	11	4	2	5	-	1	1	2	2	2	3
30	13	8	4	3	1	-	-	1	-	-	1	6
All	118	136	38	42	48	8	16	20	40	20	11	29

**Fig. 1:**
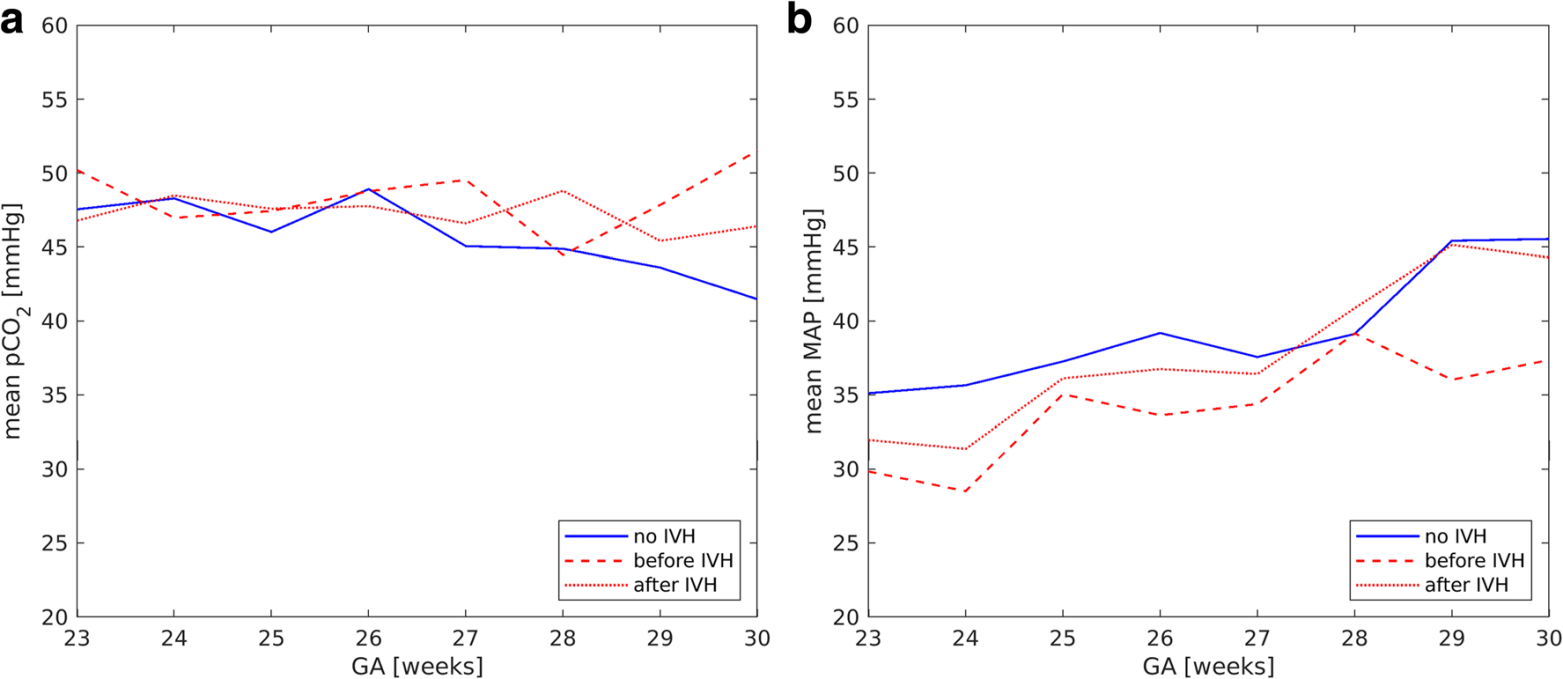
Mean value of pCO_2_ (**a**) and MAP (**b**) versus GA for the control group (blue lines) and the affected group (red lines) before (dashed lines) and after (dotted lines) IVH

Figure 1 shows the dependence of pCO_2_ and MAP on GA both for the control and the affected group before and after diagnosis of IVH.

**Fig. 2:**
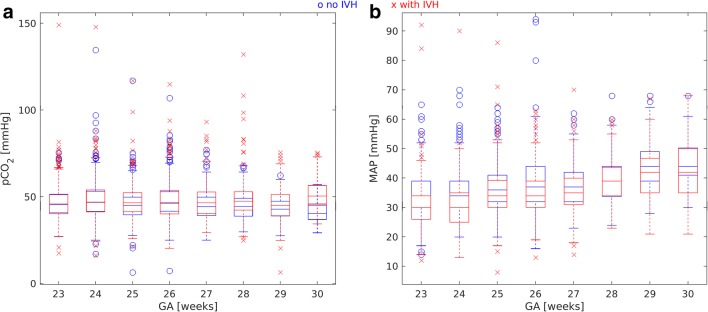
Box plots of pCO_2_ (**a**) and MAP (**b**) versus GA for the control group (blue symbols) and the affected group (red symbols)

**Fig. 3:**
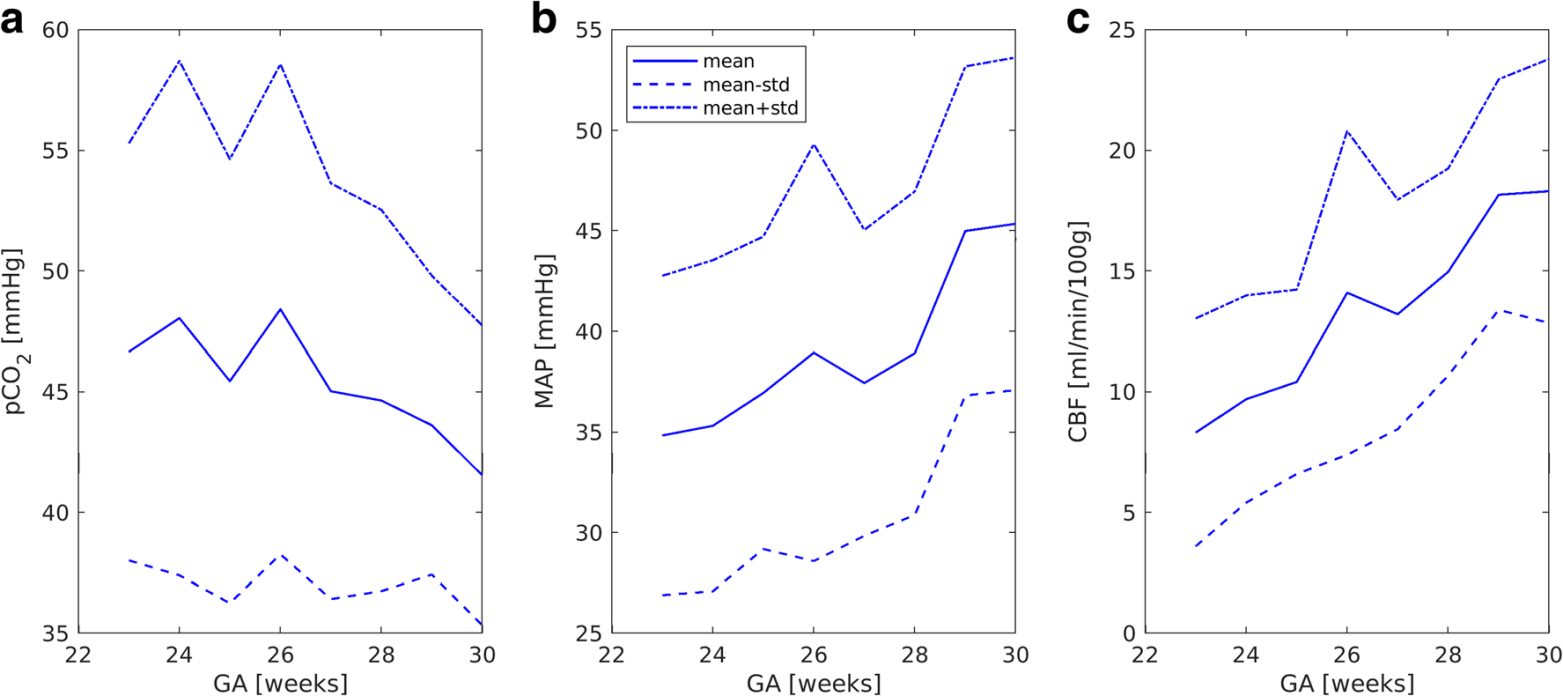
Limiting values for pCO_2_ (**a**), MAP (**b**), and CBF (**c**) versus GA

**Fig. 4:**
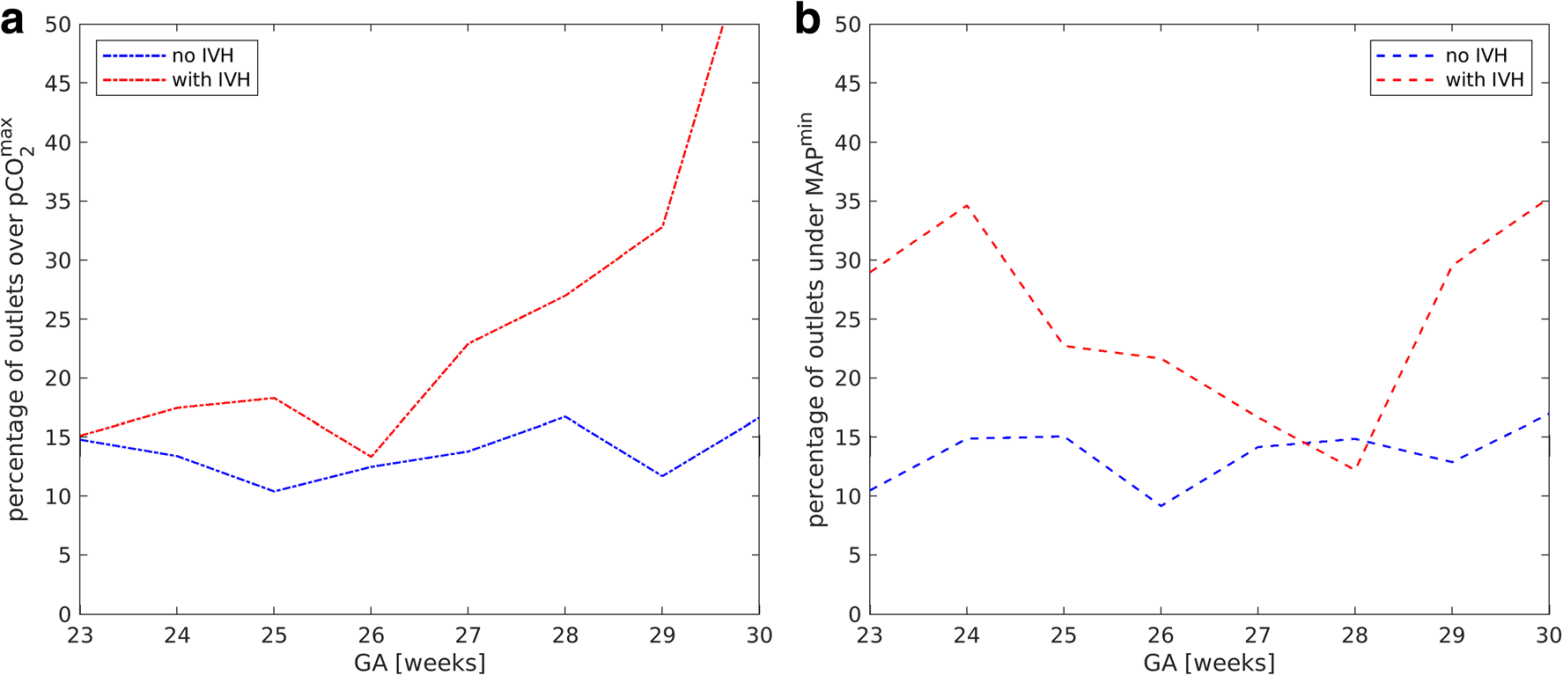
Number of outliers of pCO_2_ (**a**) and MAP (**b**) versus GA

No correlation between GA and arterial pCO_2_ was found. The mean value of arterial pCO_2_ in the affected group was significantly higher than in controls before (*p* < 0.01) and after (*p* = 0.01) IVH (Fig. 1a and Table 3). The analysis of box plot (Fig. 2a) suggests that this effect is caused by the outliers. Defining for each gestational age, the upper threshold pCO_2_^max^ as the mean value + standard deviation of the control group (Fig. 3a), we could observe (Fig. 4a) that for all gestational ages, the percentage of pCO_2_ records exceeding the upper limit pCO_2_^max^ is higher in the affected group.

**Table 3:** Mean values of clinical parameters for GA 23–30 weeks

	no IVH	before IVH	after IVH
pCO_2_ (mmHg)	46.7 ± 9.5	48.3 ± 10.7 p* < 0.01	47.6 ± 10.6 p** = 0.01
MAP (mmHg)	37.8 ± 9.0	33.6 ± 8.5 p* < 0.01	35.9 ± 8.9 p** < 0.01
CBF (ml/min/100 g)	12.2 ± 5.8	12.5 ± 7.2 p* = 0.6	13.2 ± 8.2 p** = 0.2
MAD_CBF_ (ml/min/100 g)	4.4 ± 3.7	5.7 ± 4.5 p* < 0.01	5.8 ± 5.9 p** < 0.01

^*^Comparison between “no IVH” and “before IVH” values

^**^Comparison between “no IVH” and “after IVH” values

MAP increased with increasing GA both in the control and the affected group (Pearson correlation coefficients are 0.34 and 0.43, respectively, *p* < 0.01 in both cases). Before IVH (Fig. 1b and Table 3), the mean value of MAP in the affected group was significantly lower (*p* < 0.01) than the one in the control group. After IVH (Fig. 1b and Table 3), for the affected group with GA of 23–30 weeks, MAP mean values increased, but stayed lower than in the control group. For GA of 23–26 weeks, this difference was significant (controls 36.8 ± 8.9, affected 34.0 ± 8.3, *p* < 0.01), while for GA of 27–30 weeks, mean values were statistically equal (controls 40.5 ± 8.6, affected 40.6 ± 8.8, *p* = 0.7). The role of outliers is seen in the box plot (Fig. 2b). For gestational age before 27 WG, both very high and very low values were observed for the affected group. The percentage of MAP records that were lower than the lower threshold MAP^min^ (Fig. 3b), being defined as the mean value minus standard deviation of the control group, was higher for the affected group (Fig. 4b).

**Fig. 5:**
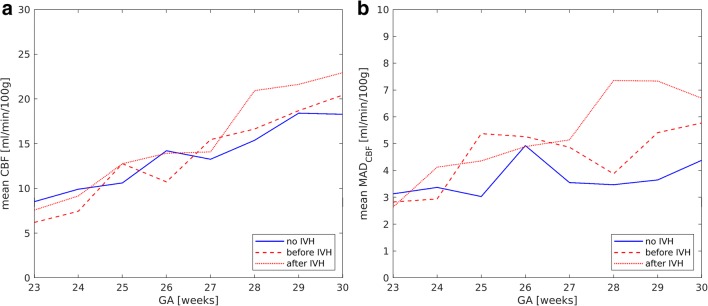
Mean value (**a**) and mean absolute deviation (**b**) of CBF versus GA for the control group (blue lines) and the affected group (red lines) before (dashed lines) and after (dotted lines) IVH

**Fig. 6:**
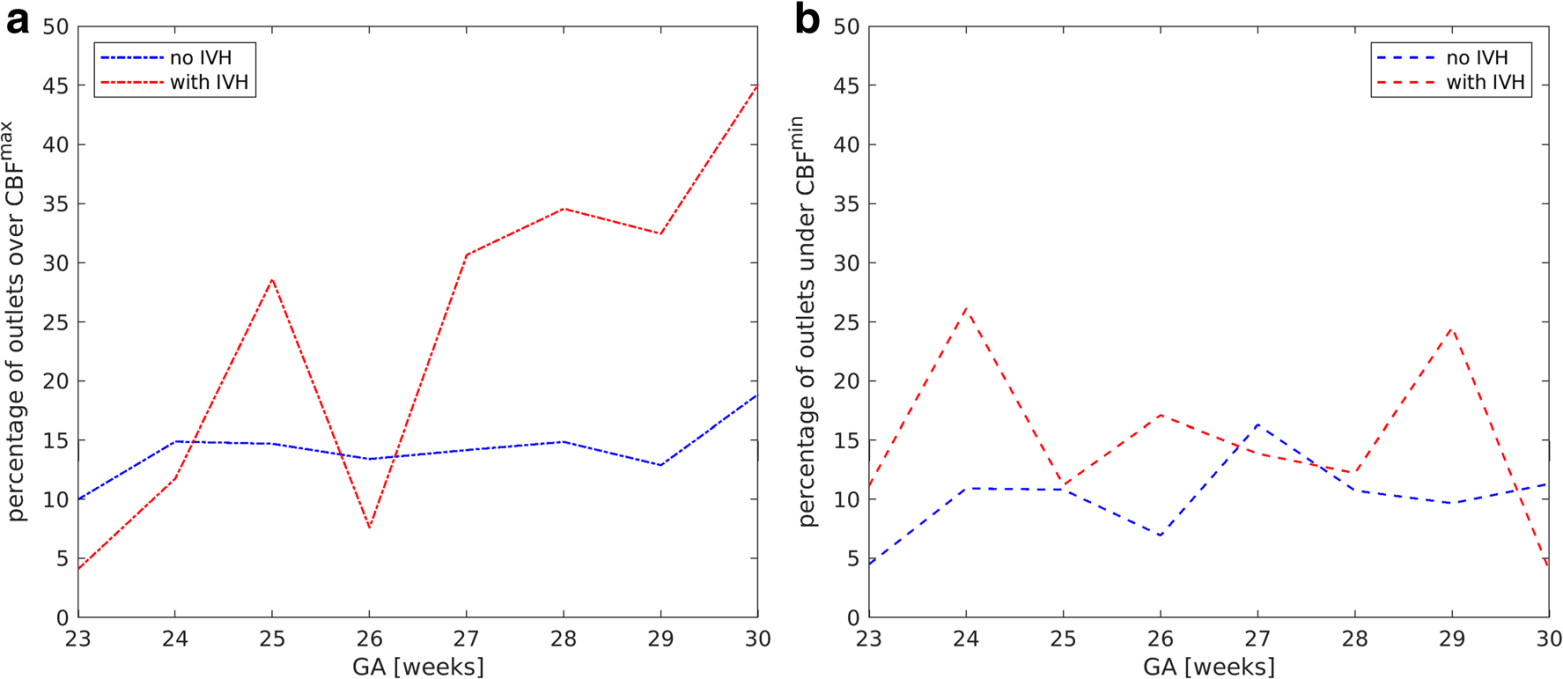
Number of outliers under (**a**) and over (**b**) limiting values of CBF versus GA

Mean CBF values, derived via mathematical modeling, increased with GA (Fig. 5a) both in the control and in the affected group (Pearson correlation coefficients are 0.6 and 0.5, respectively, *p* < 0.01 in both cases). No correlation between CBF and IVH grade was detected. Mean CBF values before and after IVH in the affected group were similar to those in the control group (Table 3), but extreme CBF values occurred more frequently in the affected group. Figure 5b shows the dependence of MAD_CBF_ on GA for the control and affected groups before and after IVH. The MAD_CBF_ in the affected group before and after IVH was significantly higher (*p* < 0.01 in both cases) than that in the control group (Table 3). The percentage of CBF values above CBF^max^ (Fig. 6a) or below CBF^min^ (Fig. 6b) was higher in the affected group. The threshold values CBF^max^ and CBF^min^ were evaluated from the control group as mean value ± standard deviation (Fig. 3c).

## Discussion

IVH remains a major cause of significant brain injury in very preterm infants despite progress in obstetric and neonatal intensive care. By comparison of medical characteristics of the affected IVH group before diagnosis with controls, risk factors for IVH can be identified. The current study aimed at assessing changes in the three most important [9] clinical parameters, before and after IVH, in comparison with reference values of the unaffected control group. While values of pCO_2_ and MAP were obtained from clinical records, values of CBF were calculated by mathematical modeling [17, 18]. This is a novel approach in the investigation of the development of IVH in very preterm infants.

The study was based on the clinical records of 254 infants with gestational age between 23 and 30 WG. The control group was matched with the affected group for GA and mean BW. It was extremely difficult to match the patients with 23 WG, because almost all preterm infants of this gestational age had IVH. However, the authors believe that the difference in the number of patients with and without IVH did not affect the results of the study because all conclusions were based on averaged and relative values (in percent).

In comparison with the control group, higher values of pCO_2_ were observed in the affected group before and after IVH. This is consistent with the potential of hypercapnia to induce IVH [21, 22], because hypercapnia produces vasodilation and leads to an increase in CBF [20].

For each gestational age, the critical upper limit pCO_2_^max^ was evaluated from the control group as a mean value + standard deviation. The percentage of pCO_2_ values exceeding pCO_2_^max^ was higher in the affected group than in the control group. These critical upper values can be proposed as thresholds above which the probability of IVH increases.

Before IVH, the mean values of MAP were significantly lower than those in the control group. Likewise, after IVH, MAP in the affected group was significantly lower than in the control group, but only for extremely preterm infants with GA of 23–26 weeks. Our results were consistent with previous observations by Al-Aweel et al. [23] made for infants at 26–28 WG, indicating a close relation between neonatal IVH and arterial hypotension, which was determined using the Versmold et al.’s standards [23, 24]. According to Bada et al. [25], neonates having IVH grade II–IV also showed significantly lower arterial MAP as compared with children without IVH or having IVH grade I. This also supports our research.

There is no consensus regarding normal blood pressure in premature neonates [26–28]; however, some studies indicate that the neuroprotective mechanism of autoregulation is lost below MAP of 30 mmHg [26]. For example, infants who developed severe IVH have been shown as having substantially more unstable MAP, and therefore, spent significantly more time with extreme MAP values (< 23 mmHg or > 46 mmHg) as compared with those without severe IVH [27]. The same tendency was observed in our study. Because of the correlation detected between MAP and GA, the threshold values in the present study, as in [27, 28], were not fixed, but rather, they were evaluated from the control group for each gestational week. The percentage of extreme MAP values was higher in the affected group than in the control one. This result is in agreement with the experimental findings of da Costa et al. [28], who established that deviations below optimal MAP were greater in the IVH group as compared with those with no IVH.

The results presented here show that calculated mean CBF values in the affected group were similar to those for the control infants of the same GA. Furthermore, for both groups, the mean CBF values rose with GA. However, significantly larger mean deviations in CBF before and after IVH were observed in the neonates of the affected group as compared with the control group. Moreover, the percentage of extreme values was also higher in the affected group than in the control one. To evaluate these extreme values, threshold limits were calculated using the CBF values of the control group. The threshold limits increased with GA. We suggest that these limits be used for routine monitoring of the probability of IVH.

The following limitations should be mentioned. All clinical data were collected retrospectively, and therefore, the number of recordings was different for each infant. Additionally, the measurement of blood gases was done according to clinical routine, which yielded mainly point records and not continuous ones. This means that there may have been variations in parameter values that have not been considered here. Furthermore, having considered only measurements for which the coincident records of pCO_2_ and MAP are available also reduced the number of available measurements for analysis. Another limitation is that the precise hemorrhage timing was not exactly known, because standard cranial ultrasound was not performed routinely every day. Lastly, only three clinical parameters, although the most important ones [9], were analyzed in our research. The origin of IVH, however, is multifactorial [3] and other medical conditions may also play a role. Nevertheless, the main conclusions of the present study could be helpful for better prediction of IVH and its evolution.

## Conclusions

The mean values of clinically measured pCO_2_ and MAP, as well as the mean absolute deviation of numerically calculated CBF, in premature neonates who developed IVH showed a statistically significant difference in the corresponding values in premature neonates without IVH. The typical characteristics of the infants diagnosed with IVH were hypercapnia (increased pCO_2_), hypotension (decreased MAP), and highly fluctuating CBF. Each of these conditions can lead to damage of the fragile vessels of the germinal matrix, and therefore, to the origin of IVH. As shown here by the quantification of CBF, future research on the processes leading to IVH can benefit from mathematical modeling of structural and metabolic conditions in the immature brain.

## Abbreviation

BW: Birth weight
CBF: Cerebral blood flow
GA: Gestational age
IVH: Intraventricular cerebral hemorrhage
MAD_CBF_: Mean absolute deviations of CBF
MAP: Mean arterial pressure
PCO_2_: Arterial carbon dioxide pressure
WG: Week of gestation
